# Latent profiles and correlates factors of cognitive function in older adults: a cross-sectional study

**DOI:** 10.3389/fnagi.2025.1622804

**Published:** 2025-09-24

**Authors:** Jin Xiang, Yan Xiong, Heting Liang, Qingyun Mao, Yumeng Zhang, Yunting Li, Zhixia Jiang, Xiaoli Yuan

**Affiliations:** ^1^Department of Nursing, Affiliated Hospital of Zunyi Medical University, Zunyi, China; ^2^Guizhou Nursing Vocational and Technical College, Guizhou, China

**Keywords:** older adults, cognitive function, latent profile analysis, correlates factors, community nursing

## Abstract

**Objective:**

This study aimed to identify the latent profiles of cognitive function among community-dwelling and institutionalized older adults, and to examine their associated influencing factors, in order to inform the development of targeted interventions.

**Methods:**

A convenience sampling method was used to select 6,708 elderly people aged 60 years and older from six communities and nine long-term care institutions across China, who were assessed using a general information questionnaire, Mini-Mental State Examination (MMSE), the Frailty Scale, the Anxiety Scale, the Depression Scale, and the Pittsburgh Sleep Quality Index. Latent profile analysis (LPA) was performed based on the MMSE scores, and multiple logistic regression was used to analyse the influencing factors of cognitive function categories.

**Results:**

A total of three cognitive function profiles were identified: High cognitive Function group (41.2%), Moderate Cognitive Function Group (48.2%) and Low cognitive Function group (10.7%). Higher Frailty [odds ratio (ORs) = 1.070–1.246], higher depressive symptom scores (OR = 1.059–1.191) and poorer sleep quality (higher PSQI; OR = 1.088) were associated with higher odds of belonging to the Moderate/Low cognitive profiles, whereas adequate social support (Yes vs. No; OR = 0.530–0.696), selected middle-income categories versus ≥¥6,000 in per-capita monthly household income (OR = 0.462–0.735) and male sex (OR = 0.556–0.876) were associated with lower odds.

**Conclusion:**

Cognitive function among older adults can be classified into three distinct latent profiles, each associated with different influencing factors. These findings underscore the need for stratified and personalized interventions at the community level to support stratified screening and tailored community programs; given the cross-sectional design, these associations do not establish causality or intervention effects.

## 1 Introduction

The United Nations, Department of Economic and Social Affairs, Population Division (2017) reported that global population aging is accelerating. By 2030, the number of people aged 60 years and older is projected to increase by 56%, from 962 million to 1.4 billion, with the fastest growth expected in developing countries (United Nations, Department of Economic and Social Affairs, Population Division, 2017). As aging intensifies, the health of older adults has become a major policy priority at national and regional levels, drawing growing attention from society and the scientific community. Cognitive decline is among the most common health issues in later life, and its prevalence is rising globally (World Health Organization, 2025; Song et al., 2023). Studies have shown that mild cognitive impairment (MCI) increases with age, affecting approximately 21.2% of the older population worldwide (Chen et al., 2023a). Cognitive deterioration not only compromises daily functioning and quality of life, but also imposes a substantial burden on families and healthcare systems (McGrath et al., 2020). In China, national strategic documents such as *National Medium-to-Long-Term Plan for Proactively Addressing Population Aging* (The Central Committee of the Communist Party of China and the State Council, 2019) and *14th Five-Year Plan for the Development of Aging Causes and the Elderly Care Service System* (The State Council of the People’s Republic of China, 2021) have prioritized the early detection and intervention of cognitive impairment. These policies emphasize strengthening community-based cognitive health services and promoting the implementation of multilevel and individualized intervention strategies. Such efforts are in line with global priorities for preventing cognitive decline. Recent research suggests that cognitive decline in older adults is not a uniform, linear process, but is instead marked by substantial individual variation (McGrath et al., 2020; Rouanet et al., 2022; Zhou et al., 2021). Even among “cognitively normal” older individuals, some exhibit subtle but persistent decline over time (Tiantian et al., 2022). Existing research further indicates that changes in cognitive functioning and resilience are associated with a variety of psychosocial and physiological factors, including depression (Yin et al., 2024; Shimada et al., 2014), social support and social participation (Kelly et al., 2017; Mogic et al., 2023), marital status (Liu et al., 2019), sleep quality (Liao et al., 2022), frailty (Ma et al., 2019), gender (Delpak and Talebi, 2020), and socioeconomic resources (Rodriguez et al., 2021), and that these factors may operate jointly in ways that these psychosocial and physiological factors are intertwined in a manner that erodes cognitive reserve and physiological resilience. Collectively, these variables were chosen because they are consistently reported and potentially modifiable risk or protective factors for cognitive aging. Considering them together helps capture the multidimensional interplay that shapes cognitive reserve and resilience. Socioeconomic disadvantage and social participation are not only direct stressors, but also have significant indirect negative effects on cognitive functioning through intermediate links that lead to depression and sleep disturbances (Holland et al., 2024), at the same time, frailty is widely recognized to be strongly associated with cognitive performance decline (Dong et al., 2024). However, most existing studies adopt a variable-centered analytic paradigm, focusing on estimating the average net effects of individual covariates on cognitive outcomes, and devote relatively little attention to identifying person-centered, multidimensional patterns of within-person covariation (Van Lissa et al., 2024). Although a number of recent LPA-based studies have shown that distinguishable cognitive profiles exist in older adults, evidence remains limited in specific populations (e.g., community- and institution-dwelling older adults) that use MMSE domain-level indicators to characterize cognitive combinations and, within a single framework, simultaneously examine the associated factors of depression, sleep, frailty, social support/marital status, social participation, sex, and socioeconomic resources (Dong et al., 2024). In this context, this study employs latent profile analysis (LPA), based on the five cognitive dimension scores of the MMSE, to identify potential subcategories of cognitive functioning in the Chinese elderly population. By systematically examining multidimensional factors associated with different cognitive profiles in a cross-sectional design, this study aims to provide empirical evidence for community and primary health care providers to develop accurate screening and individualized intervention strategies, to inform early identification and service planing, without implying causal effects. Ultimately, these associations are intended to support strategies that may help delay cognitive decline, enhance quality of life in later life, and reduce the societal burden of care.

## 2 Materials and methods

### 2.1 Participants

Data for this study were derived from the “Key Special Project on Active Health and Technological Response to Aging” under China’s National Key Research and Development Program (Project No. 2020YFC2008500). Between October 2022 and September 2023, a total of 6,708 individuals aged 60 years and older were recruited using convenience sampling from six grassroots community centers and nine independent long-term care institutions across China. Of these participants, 6,394 (95.3%) were community-dwelling older adults and 314 (4.8%) were institutionalized residents. Inclusion criteria were: (1) aged ≥60 years; and (2) voluntary participation with signed informed consent. Exclusion criteria included: (1) a history of severe psychiatric illness or communication disorders; (2) acute critical medical conditions (e.g., shock, respiratory failure, acute heart failure, acute myocardial infarction, or stroke); and (3) acute exacerbation of chronic disease or terminal-stage illness with an expected survival of less than 3 months. The study protocol was approved by the Ethics Committee of the Affiliated Hospital of Zunyi Medical University (Approval No: KLL-2022-814) and conducted in accordance with the ethical principles outlined in *Declaration of Helsinki*. All participants provided written informed consent prior to enrollment.

### 2.2 Survey instruments

1. General Information Questionnaire: Developed by the research team based on a review of relevant literature, this questionnaire included two major sections. The first covered socio-demographic variables such as gender, age, education level, marital status, residence location, living arrangement, per capita household income, and social support. The second section assessed health-related behaviors, including smoking, alcohol consumption, physical activity, social engagement, and chronic disease status.
2. The Chinese Mini-Mental Status (CMMS): The Chinese Mini-Mental Status (CMMS) consists of 30 questions with a total score of 30, assessing time orientation (5 points), place orientation (5 points), transient memory (3 points), attention and calculation (5 points), delayed recall (3 points), language function (8 points), and visuospatial ability (1 point). Higher scores indicate better cognitive functioning. The results of this scale should be judged in relation to the level of education: a total score of ≤17 in the illiterate group, ≤20 in the elementary school group, and ≤24 in the junior high school and above group is considered to have impaired cognitive functioning, The Cronbach’s alpha coefficient for CMMS in this study was0.83 (Zhang et al., 1990).
3. Proposed by Fried et al. (2001), the Fried frailty phenotype has been widely applied in both cross-sectional and longitudinal studies.this scale assesses physical frailty based on five criteria: unintentional weight loss, exhaustion, low physical activity, slow gait speed, and weak grip strength. Each component is scored as present or absent, yielding a total score ranging from 0 to 5. A score of 0 indicates non-frail, 1–2 indicates pre-frail, and ≥3 indicates frail.
4. Geriatric Depression Scale – 15 items (GDS-15): Originally developed by Yesavage et al. (1982) and later refined by Sheikh et al. (1991), the GDS-15 consists of 15 yes/no items. Each positive response scores 1 point, resulting in a total score ranging from 0 to 15. Higher scores indicate more severe depressive symptoms. All of this scale showed high internal consistency and the Cronbach’s alpha coefficient for this scale was 0.758 (Zhang et al., 2020).
5. Self-Rating Anxiety Scale (SAS): Developed by Zung et al. (1971), the SAS is used to assess subjective anxiety symptoms. The Chinese version of the scale is widely adopted and has demonstrated good reliability and validity (Xinxu et al., 2024). The scale consists of 20 items rated on a 4-point Likert scale. Scores are converted to standardized scores, with a cut-off of ≥50 indicative of clinically significant anxiety. Higher scores reflect more severe anxiety levels. The results of the study show that SAS has a strong internal consistency coefficient of 0.80 (Ramirez and Lukenbill, 2008). The Cronbach’s alpha coefficient for the Chinese version of SAS in this study was 0.78 (Pang et al., 2019).
6. Pittsburgh Sleep Quality Index (PSQI): Developed by Buysse et al. (1989), the PSQI is used to assess subjective sleep quality over the past month. The Chinese version has shown good reliability and validity (Taoying et al., 2014). It includes 19 self-rated items across seven components. Each component is scored from 0 to 3 (component scores 0–3), and the total score ranges from 0 to 21. Higher total scores reflect poorer sleep quality. The reliability of the PSQI in this study was 0.994. The split-half reliability coefficient of the PSQI was 0.824 and the overall Cronbach′s alpha coefficient was 0.845 (Taoying et al., 2014).

### 2.3 Survey methods and quality control

The field survey was conducted by a team of master’s-level nursing students who had received standardized training. Investigators explained the study objectives and questionnaire completion requirements to participants and provided guidance using a standardized script. For participants unable to complete the questionnaire independently, assistance was provided by trained personnel. All questionnaires were collected immediately after completion and reviewed on-site for completeness and accuracy. Any missing responses were promptly corrected to ensure high data quality.

## 3 Statistical methods

Data were analyzed using SPSS version 29.0 and Mplus version 8.3. In Mplus, latent profile analysis (LPA) was constructed using z standard scores of the five dimensions of MMSE as the observables. LPA belongs to the modeled clustering method of finite mixture models, which explains the covariance of a set of continuous indicators by introducing categorical latent variables and completes the assignment of individuals based on a posteriori probabilities. The class size is determined by a combination of Akaike Information Criterion (AIC), Bayesian Information Criterion (BIC), sample-size adjusted BIC (aBIC), entropy, Lo–Mendell–Rubin adjusted likelihood ratio test (LMRT), and the Bootstrap likelihood ratio test (BLRT), which trade off between goodness-of-fit and substantive interpretability [see Nylund et al. (2007) and Tein et al. (2013)]. After fitting the class 1–5 model, the optimal solution was determined by combining the above metrics with interpretability. Both descriptive and inferential statistics were completed based on raw scores. Near-normal continuous variables are expressed as mean ± standard deviation; skewed distributions are expressed as median (interquartile range); and categorical variables are expressed as number of cases (%). For baseline continuous covariates that deviated from normality, the Kruskal-Wallis H test was used for between-group comparisons; the Chi-square (χ^2^) test was used for categorical variables. For comparisons of MMSE dimension means between potential categories, homoscedasticity was assessed with Levene’s test. When violated, we used Welch’s one-way ANOVA with Games–Howell pairwise contrasts; when satisfied, classical one-way ANOVA with Tukey Honestly Significant Difference (HSD). Eta-squared (η^2^) was reported as an effect size and statistical significance was defined as two-sided *α* = 0.05. Variables that were significant in univariate analyses (*p* < 0.05) were entered into the multinomial logistic regression. To address multiplicity, we applied Holm–Bonferroni to the across-domain omnibus tests; and applied Benjamini-Hochberg False Discovery Rate (BH-FDR) (*q* = 0.05) across other omnibus comparisons. In the multinomial models, prespecified key predictors were anxiety, sleep, depression, and frailty, and their *p* values were Holm–Bonferroni adjusted (*m* = 4); other covariates were treated as adjustments with ORs and 95% confidence intervals (CI) reported.

## 4 Result

### 4.1 Characteristics of the sample of community-dwelling older adults

A total of 6,708 cases of elderly people were investigated in this study, including 3,264 (48.7%) males and 3,444 (51.3%) females, with the age range of 60–98 years old, with an average age of 71.44 ± 7.33 years old, of which the number of people aged 60–69 years old was 2,957, which accounted for the largest proportion (44.1%); Han Chinese accounted for the majority of the ethnic groups, with a total of 6,043 people (90.1%) Among the ethnic groups, Han Chinese accounted for the majority, with 6,043 (90.1%), and 665 (9.9%) of other ethnic groups. In terms of literacy, the largest number of participants were uneducated, totaling 2,865 (42.7%), followed by 1,689 (25.2%) in elementary school, 1,296 (19.3%) in junior high school, 611 (9.1%) in high school/secondary/technical school, and 217 (3.2%) in junior college and above. Married people were in the majority, totaling 5,579 (83.2%), while unmarried/divorced/widowed totaled 1,129 (16.8%). The mode of residence was 398 (5.9%) living alone and 6,310 (94.1%) living together. The general demographic characteristics of the study population are shown in Table 1.

**Table 1:** Sociodemographic and health-related characteristics of community-dwelling older adults (*n* = 6,708).

Variable	Category	*n* (%)
Sociodemographic characteristics		
Gender	Female	3,444 (51.3)
	Male	3,264 (48.7)
Age (years)	60–69	2,957 (44.1)
	70–79	2,704 (40.3)
	80–89	971 (14.5)
	90–99	76 (1.1)
Ethnicity	Han	6,043 (90.1)
	Other	665 (9.9)
Education level	No formal education	2,865 (42.7)
	Primary school	1,689 (25.2)
	Junior high school	1,296 (19.3)
	Senior high school / Vocational school	611 (9.1)
	College degree or above	217 (3.2)
Marital status	Married	5,579 (83.2)
	Unmarried/Divorced/Widowed	1,129 (16.8)
Living arrangement	Living alone	398 (5.9)
	Living with others	6,310 (94.1)
Health-related characteristics		
Per-capita monthly household income (¥)	<2000	3,410 (50.8)
	2000–2,999	1,358 (20.2)
	3,000–3,999	793 (11.8)
	4,000–4,999	419 (6.2)
	5,000–5,999	359 (5.4)
	≥6,000	369 (5.5)
Chronic diseases	No	4,973 (74.1)
	Yes	1735 (25.9)
Alcohol consumption	No	5,656 (84.3)
	Yes	1,052 (15.7)
Smoking	No	5,335 (79.6)
	Yes	1,373 (20.5)
Social participation	No	2,491 (37.1)
	Yes	4,217 (62.9)
Regular exercise	No	1,617 (24.1)
	Yes	5,091 (75.9)
Adequate social support	No	783 (11.7)
	Yes	5,925 (88.3)

### 4.2 Scores of MMSE dimensions among community-dwelling older adults

In this study, a total of 6,708 older adults in six communities and nine nursing homes in Zunyi City, Guizhou Province, China, were surveyed with questionnaires. The results showed that the overall score of MMSE was 21.99 ± 5.999. The scores according to the five dimensions in the MMSE and the overall score are shown in Table 2.

**Table 2:** MMSE assessment results among community-dwelling older adults (*n* = 6,708).

Domain	Number of items	Score range	Score (Mean ± SD)
Orientation	10	0–10	8.57 ± 1.951
Memory	3	0–3	2.54 ± 0.781
Attention and calculation	5	0–5	2.62 ± 1.821
Recall	3	0–3	1.85 ± 1.050
Language ability	9	0–9	6.40 ± 2.037
MMSE total score	30	0–30	21.99 ± 5.999

### 4.3 Latent profile analysis of cognitive function in older adults

Using the standardized scores of the five MMSE dimensions as observed indicators, latent profile models with one to five classes were constructed. Model fit indices are presented in Table 3. As the number of latent classes increased, values of AIC, BIC, and aBIC gradually decreased. All models yielded significant *p*-values (< 0.01) for both the LMRT and the BLRT, indicating statistically significant improvements in model fit. Entropy values for all models exceeded 0.80, suggesting high classification accuracy. The three-class model achieved an entropy of 0.891, indicating strong classification quality. Although the entropy values of the four-class and five-class models were slightly higher (0.908 and 0.919, respectively), these models were more complex and offered limited clinical interpretability. Based on both statistical fit and substantive interpretability, the three-class model was selected as the optimal solution.

**Table 3:** Fit indices for latent profile models of cognitive function in older adults.

Model	AIC	BIC	aBIC	LMRT (p)	BLRT (p)	Entropy	Class membership probability				
1	95197.396	95265.507	95233.729								
2	86182.857	86291.834	86240.990	0.0000	0.0000	0.823	0.30352	0.69648			
3	81728.545	81878.388	81808.478	0.0000	0.0000	0.891	0.41234	0.48241	0.10525		
4	75530.575	75721.284	75632.307	0.0000	0.0000	0.908	0.11643	0.26479	0.19961	0.41917	
5	74605.098	74836.674	74728.631	0.0000	0.0000	0.919	0.11643	0.14177	0.05784	0.30620	0.37776

Fit indices for latent profile models of cognitive function in older adults.

Entropy indicates classification accuracy (range: 0–1); higher values reflect better separation between classes.

Class membership probability refers to the estimated proportion of participants assigned to each latent class.

### 4.4 Naming of cognitive function profiles

Based on the standardized mean scores of the five MMSE dimensions, a latent profile analysis was conducted, classifying participants into three cognitive function profiles.

Class 1 comprised 41.2% of participants (*n* = 2,766), with the highest scores across all cognitive domains, indicating superior cognitive performance; this class was labeled the “High Cognitive Function Group.” Class 2 included 48.2% of participants (*n* = 3,236), characterized by relatively low scores in the attention and calculation domain and moderate performance in other domains; this group was labeled the “Moderate Cognitive Function Group.”

Class 3 accounted for 10.7% of participants (*n* = 706) and exhibited consistently low scores across all cognitive domains, representing the lowest cognitive performance; this class was labeled the “Low Cognitive Function Group.”

**Figure 1:**
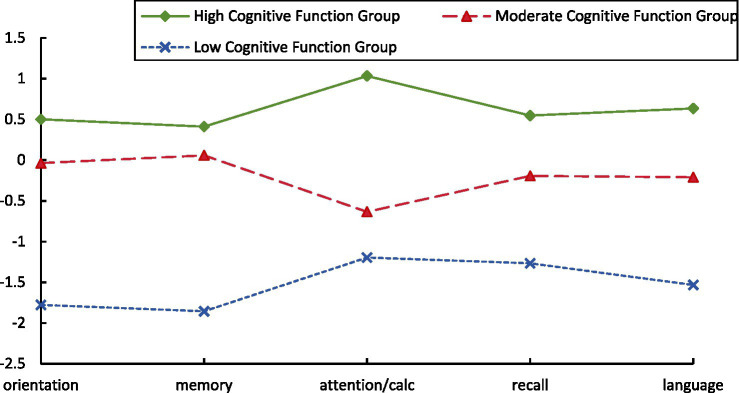
Distribution of latent cognitive profiles among older adults. Standardized cognitive dimension scores across latent cognitive function profiles in older adults. The figure illustrates the distribution of five MMSE subdomain scores (orientation, memory, attention and calculation, recall, and language) across the three latent cognitive function groups identified via latent profile analysis: high (green), moderate (red), and low (blue). Scores were standardized (*Z*-scores) to allow comparability across dimensions. Higher values indicate better cognitive performance.

Figure 1 displays the cognitive profile trajectories for the three classes.

Differences in profiles across potential categories on MMSE dimensions. Using the raw scores of the five MMSE dimensions as indicators, the three profiles showed a clear and interpretable pattern: the High Cognitive Function Group performed best across all dimensions; the medium cognition group was generally intermediate, but showed significant “sink” in the attention and calculation dimensions; and the Low Cognitive Function Group was significantly impaired in all dimensions, especially orientation and delayed recall. Levene’s tests indicated heteroscedasticity across MMSE domains (Levene *p* < 0.001), therefore, Welch’s ANOVA with Games–Howell pairwise comparisons was used.robust Welch’s tests showed significant overall differences in all dimensions (all *p* < 0.001), and two-by-two comparisons (Games-Howell) showed all pairwise comparisons to be statistically significant (all *p* < 0.001). In terms of effect sizes, Attention and Computation had the largest differentiation (*η*^2^ = 0.805), while Orientation, Memory Registration and Verbal-Execution also had large effect sizes (*η*^2^ ≈ 0.44–0.46), and Delayed Recall was a medium effect (*η*^2^ = 0.324). In terms of within-class variation, the Moderate Cognitive Function Group had the largest dispersion on Attention and Computation [standard deviation (SD) = 0.88], while the remaining dimensions had more moderate dispersion within each category (see Table 4). After Holm adjustment across the five MMSE domains, all omnibus tests remained significant (all *p*adj < 1 × 10^−300^). Within-domain pairwise comparisons using Games–Howell consistently showed High > Moderate > Low.

**Table 4:** MMSE domain scores by latent profiles (Mean ± SD).

Domain	High (*n* = 2,766)	Moderate (*n* = 3,236)	Low (n = 706)	Omnibus *p* (Welch)	Effect size (η^2^)	Pairwise (Games–Howell)
Orientation (0–10)	9.55 ± 0.96	8.50 ± 1.51	5.08 ± 2.47	<0.001	0.442	High>Moderate>Low (all *p* < 0.001)
Registration/Immediate memory (0–3)	2.86 ± 0.43	2.58 ± 0.61	1.05 ± 0.85	<0.001	0.456	High>Moderate>Low (all *p* < 0.001)
Attention–Calculation (0–5)	4.54 ± 0.72	1.46 ± 0.88	0.42 ± 0.71	<0.001	0.805	High>Moderate>Low (all p < 0.001)
Recall/Delayed memory (0–3)	2.44 ± 0.84	1.65 ± 0.92	0.49 ± 0.69	<0.001	0.324	High>Moderate>Low (all p < 0.001)
Language and praxis (0–9)	7.71 ± 1.29	5.98 ± 1.62	3.24 ± 1.84	<0.001	0.444	High>Moderate>Low (all p < 0.001)

High (class = 3) *n* = 2,766; Moderate (class = 1) *n* = 3,236; Low (class = 2) *n* = 706.

Raw scores; higher scores indicate better cognition. Levene’s tests indicated heteroscedasticity across domains (all *p* < 0.001); therefore, omnibus differences are reported from Welch’s ANOVA; pairwise comparisons use Games–Howell; *η*^2^ from ANOVA effect size. Abbreviations: SD, standard deviation; MMSE, Mini-Mental State Examination. Across-domain omnibus *p* values (five tests) were Holm–Bonferroni adjusted; within-domain pairwise comparisons used Games–Howell.

### 4.5 Comparison of general characteristics and univariate analysis across cognitive function profiles

Levene’s tests indicated heteroscedasticity across groups for continuous variables (*p* < 0.05); therefore, Welch’s ANOVA with Games–Howell *post hoc* tests was applied, while categorical variables were compared using Chi-square tests. General characteristics were compared among older adults classified into the three latent cognitive function profiles. The results indicated statistically significant differences (*p* < 0.05) across groups in gender, age, ethnicity, marital status, education level, social engagement, monthly household income, presence of chronic diseases, alcohol consumption, smoking status, regular exercise, Adequate social support, frailty score, depression score, anxiety score, and PSQI total score.

No significant difference was found in living arrangement among the three groups (*p* > 0.05). Detailed results are presented in Table 5. After controlling multiplicity across ~15 omnibus tests using BH-FDR (*q* = 0.05), the overall significance pattern was unchanged (all *p* adj ≤ 0.05).

**Table 5:** Univariate analysis of general characteristics across latent cognitive function profiles in 6,708 older adults.

Variable	Category	High cognitive function group (*n* = 2,766)	Moderate cognitive function group (*n* = 3,236)	Low cognitive function group (*n* = 706)	*X^2^/Z*	*P*
Socio-demographic characteristics						
Gender [*n* (%)]	Male	1,570 (56.8)	1,450 (44.8)	244 (34.6)	148.048	<0.001
	Female	1,196 (43.2)	1786 (55.2)	462 (65.4)		
Age [*n* (%)]	60–69	1,544 (55.8)	1,271 (39.3)	142 (20.1)	459.733	<0.001
	70–79	966 (34.9)	1,412 (43.6)	326 (46.2)		
	80–89	238 (8.6)	518 (16.0)	215 (30.5)		
	90–99	18 (0.7)	35 (1.1)	23 (3.3)		
Ethnicity [*n* (%)]	Han	2,447 (88.5)	2,958 (91.4)	638 (90.4)	14.524	<0.001
	Other	319 (11.5)	278 (8.6)	68 (9.6)		
Education level [*n* (%)]	No formal education	737 (26.6)	1,626 (50.2)	502 (71.1)	937.275	<0.001
	Primary school	651 (23.5)	894 (27.6)	144 (20.4)		
	Junior high school	770 (27.8)	489 (15.1)	37 (5.2)		
	Senior high school / Vocational school	434 (15.7)	157 (4.9)	20 (2.8)		
	College degree or above	174 (6.3)	40 (1.2)	3 (0.4)		
Marital status [*n* (%)]	Married	2,439 (88.2)	2,646 (81.8)	494 (70.0)	141.960	<0.001
	Unmarried/Divorced/Widowed	327 (11.8)	590 (18.2)	212 (30.0)		
Living arrangement [*n* (%)]	Living alone	150 (5.4)	194 (6.0)	54 (7.6)	5.035	0.081
	Co-residing	2,616 (94.6)	3,042 (94.0)	652 (92.4)		
Per-capita monthly household income [*n* (%)]	<¥2000	1,064 (38.5)	1893 (58.5)	453 (64.2)	305.168	<0.001
	¥2000 ~ 2,999	587 (21.2)	626 (19.3)	145 (20.5)		
	¥3,000 ~ 3,999	431 (15.6)	316 (9.8)	46 (6.5)		
	¥4,000 ~ 4,999	273 (9.9)	126 (3.9)	20 (2.8)		
	¥5,000 ~ 5,999	213 (7.7)	125 (3.9)	21 (3.0)		
	≥¥6,000	198 (7.2)	150 (4.6)	21 (3.0)		
Health-related behaviors						
Presence of chronic disease [*n* (%)]	No	2097 (75.8)	2,405 (74.3)	471 (66.7)	24.399	<0.001
	Yes	669 (24.2)	831 (25.7)	235 (33.3)		
Alcohol consumption [*n* (%)]	No	2,179 (78.8)	2,849 (88.0)	628 (89.0)	109.580	<0.001
	Yes	587 (21.2)	387 (12.0)	78 (11.0)		
Smoking status [*n* (%)]	No	2060 (74.5)	2,683 (82.9)	592 (83.9)	74/233	<0.001
	Yes	706 (25.5)	553 (17.1)	114 (16.1)		
Social engagement [*n* (%)]	No	875 (31.6)	1,229 (38.0)	387 (54.8)	131.381	<0.001
	Yes	1891 (62.9)	2007 (62.0)	319 (45.2)		
Regular physical activity [*n* (%)]	No	598 (21.6)	769 (23.8)	250 (35.4)	58.871	<0.001
	Yes	2,168 (78.4)	2,467 (76.2)	456 (64.6)		
Adequate social support [*n* (%)]	No	323 (11.7)	355 (11.0)	105 (14.9)	8.560	0.014
	Yes	2,443 (88.3)	2,881 (89.0)	601 (85.1)		
Frailty score*	Raw score	0.55 ± 1.034	0.78 ± 1.169	1.40 ± 1.541	107.866	<0.001
Depression score*	Raw score	3.11 ± 2.178	3.47 ± 2.387	4.69 ± 3.271	80.882	<0.001
Anxiety score*	Raw score	42.55 ± 9.735	42.31 ± 9.893	44.66 ± 10.104	16.073	<0.001
PSQI total score*	Raw score	7.43 ± 3.170	8.34 ± 3.362	8.94 ± 3.492	86.129	<0.001

Categorical variables were compared using the Chi-square test; continuous variables were analyzed using the Kruskal–Wallis H test. PSQI, Pittsburgh Sleep Quality Index. Values for continuous variables are presented as mean ± standard deviation.

*Indicates continuous variables. *p* < 0.05 was considered statistically significant.

Omnibus *p* values across ~15 tests were adjusted using BH-FDR (*q* = 0.05).

### 4.6 Multivariate analysis of cognitive function profiles in older adults

Based on the results of the univariate analysis, a multinomial logistic regression was conducted to identify factors associated with membership in different cognitive function profiles. The three latent cognitive classes identified by latent profile analysis were treated as the dependent variable, with the high cognitive function group serving as the reference category.

Independent variables that were statistically significant (*p* < 0.05) in the univariate analysis were entered into the model. Categorical variables were dummy-coded according to their type, while continuous variables—such as frailty score, depression score, anxiety score, and PSQI total score—were entered using raw values. The detailed coding scheme is presented in Table 6.

**Table 6:** Coding scheme for independent variables.

Variable	Value assignment
Gender	0 = Male1 = Female
Age	1 = 60 ~ 692 = 70 ~ 793 = 80 ~ 894 = 90 ~ 99
Ethnicity	0 = Han1 = Other
Education level	1 = No formal education2 = Primary school3 = Junior high school4 = Senior high school / Vocational school5 = College degree or above
Marital status	0 = Unmarried / Divorced / Widowed1 = Married
Per-capita monthly household income (¥)	0 = <¥20001 = ¥2000–2,9992 = ¥3,000–3,9993 = ¥4,000–4,9994 = ¥5,000–5,9995 = ≥¥6,000
Presence of chronic disease	0 = No1 = Yes
Alcohol consumption	0 = No1 = Yes
Smoking status	0 = No1 = Yes
Social engagement	0 = No1 = Yes
Regular physical activity	0 = No1 = Yes
Adequate social support	0 = No1 = Yes
Frailty score	Raw score
Depression score	Raw score
Anxiety score	Raw score
PSQI total score	Raw score

Coding and value assignment of independent variables. Continuous variables were included using raw scores. Income is in Chinese Yuan (¥). PSQI, Pittsburgh Sleep Quality Index.

Results showed that, compared with the high cognitive function group, both the moderate and low cognitive function groups were significantly associated with multiple factors (*p* < 0.05). Detailed results of the multivariate analysis are shown in Table 7. Some education contrasts showed wide CI, indicating imprecise estimates, largely due to sparse cells from the rare ≥college reference group; these estimates should be interpreted with caution. After Holm adjustment (m = 4) applied to the prespecified predictors (anxiety, sleep quality, depression, and frailty), the significance pattern was unchanged. Consistent with the events per variable (EPV ≥ 10) rule of thumb, the smallest outcome class (n = 706) and the number of parameters per logit (≈24) yielded EPV ≈ 29 for the Low class and EPV ≈ 135 for the Moderate class, both exceeding recommended thresholds; therefore, overfitting risk was low. No concerning multicollinearity was observed [all variance inflation factor (VIF) < 5].

**Table 7:** Multinomial logistic regression analysis of latent cognitive function profiles in older adults.

Dependent variable	Independent variable	*B*	*SE*	Wald χ*^2^*	*P*	*OR* (95% *CI*)
Moderate cognitive function group	Intercept	−1.412	0.422	11.171	<0.001	
	Frailty score	0.072	0.031	5.408	0.020	1.075 (1.011–1.142)
	Depression score	0.057	0.015	14.244	<0.001	1.059 (1.028–1.091)
	Standardized anxiety score	−0.021	0.003	37.866	<0.001	0.979 (0.973–0.986)
	PSQI total score	0.085	0.010	74.465	<0.001	1.089 (1.067–1.110)
	Gender (Male vs. Female)	−0.131	0.065	4.051	0.044	0.877 (0.771–0.997)
	Age 60-69 (ref:90–99)	−0.662	0.315	4.412	0.036	0.516 (0.278–0.957)
	Age 70-79	0.330	0.314	1.103	0.294	0.719 (0.388–1.331)
	Age 80-89	0.111	0.321	0.119	0.730	1.117 (0.595–2.097)
	Ethnicity (Han vs. Other)	0.394	0.096	16.952	<0.001	1.482 (1.229–1.788)
	No formal education (ref: ≥ college)	2.080	0.197	111.516	<0.001	8.001 (5.439–11.769)
	Primary school	1.612	0.196	67.860	<0.001	5.014 (3.417–7.358)
	Junior high school	0.959	0.194	24.575	<0.001	2.610 (1.786–3.814)
	Senior high / Vocational school	0.395	0.204	3.749	0.053	1.485 (0.995–2.214)
	Marital status (Unmarried vs. Married)	0.107	0.083	1.636	0.201	1.113 (0.945–1.311)
	Social engagement (No vs. Yes)	0.093	0.069	1.809	0.179	1.097 (0.959–1.256)
	Physical activity (No vs. Yes)	−0.122	0.078	2.413	0.120	0.896 (0.759–1.032)
	Social support (No vs. Yes)	−0.363	0.094	14.909	<0.001	0.696 (0.579–0.837)
	Alcohol consumption (No vs. Yes)	0.275	0.087	10.093	0.001	1.320 (1.112–1.567)
	Smoking status (No vs. Yes)	0.194	0.083	5.491	0.015	1.214 (1.032–1.428)
	Monthly income<¥2000 (ref:≥¥6,000)	−0.027	0.140	0.038	0.846	0.973 (0.740–1.280)
	Monthly income 2000 ~ 2,999	−0.311	0.145	4.621	0.032	0.733 (0.552–0.973)
	Monthly income 3000 ~ 3,999	−0.548	0.150	13.359	<0.001	0.578 (0.431–0.776)
	Monthly income 4000 ~ 4,999	−0.769	0.169	20.787	<0.001	0.463 (0.333–0.645)
	Monthly income 5000 ~ 5,999	−0.317	0.172	3.421	0.064	0.728 (0.520–1.019)
	Chronic disease (No vs. Yes)	0.079	0.068	1.349	0.245	1.082 (0.947–1.237)
Low cognitive function group	Intercept	−3.170	0.799	15.720	<0.001	
	Frailty score	0.218	0.045	23.693	<0.001	1.244 (1.139–1.358)
	Depression score	0.175	0.022	65.351	<0.001	1.191 (1.142–1.243)
	Standardized anxiety score	−0.023	0.006	16.719	<0.001	0.977 (0.967–0.988)
	PSQI total score	0.084	0.016	28.5437	<0.001	1.088 (1.055–1.122)
	Gender (Male vs. Female)	−0.587	0.114	26.381	<0.001	0.556 (0.445–0.696)
	Age60-69 (ref:90–99)	−2.025	0.374	29.274	<0.001	0.132 (0.063–0.275)
	Age70-79	−1.179	0.367	10.311	0.001	0.308 (0.150–0.632)
	Age80-89	−0.284	0.373	0.582	0.446	0.752 (0.362–1.563)
	Ethnicity (Han vs. Other)	0.296	0.158	3.490	0.062	1.344 (0.986–1.834)
	No formal education (ref: ≥ college)	3.212	0.611	27.671	<0.001	24.831 (7.503–82.178)
	Primary school	2.214	0.612	13.102	<0.001	9.157 (2.760–30.374)
	Junior high school	0.973	0.624	2.428	0.119	2.645 (0.778–8.475)
	Senior high / Vocational school	0.877	0.643	1.860	0.173	2.403 (0.682–8.475)
	Marital status (Unmarried vs. Married)	0.454	0.118	14.892	<0.001	1.575 (1.251–1.984)
	Social engagement (No vs. Yes)	0.631	0.111	32.186	<0.001	1.880 (1.512–2.338)
	Physical activity (No vs. Yes)	−0.146	0.120	1.463	0.226	0.865 (0.683–1.094)
	Social support (No vs. Yes)	−0.635	0.148	18.336	<0.001	0.530 (0.396–0.709)
	Alcohol consumption (No vs. Yes)	0.135	0.159	0.724	0.395	1.145 (0.839–1.562)
	Smoking status (No vs. Yes)	−0.106	0.149	0.504	0.478	0.900 (0.672–1.204)
	Monthly income<¥2000 (ref:≥¥6,000)	0.090	0.282	0.101	0.750	1.094 (0.629–1.902)
	Monthly income 2000 ~ 2,999	−0.060	0.290	0.043	0.836	0.942 (0.533–1.664)
	Monthly income 3000 ~ 3,999	−0.627	0.316	3.950	0.047	0.534 (0.288–0.991)
	Monthly income 4000 ~ 4,999	−0.594	0.359	2.742	0.098	0.552 (0.273–1.115)
	Monthly income 5000 ~ 5,999	0.118	0.359	0.107	0.743	1.125 (0.557–2.274)
	Chronic disease (No vs. Yes)	−0.017	0.108	0.025	0.874	0.983 (0.796–1.215)

Multinomial logistic regression analysis of factors associated with moderate and low cognitive function profiles (reference = high cognitive function group). B, regression coefficient; SE, standard error; Reference categories: female (gender), age 90–99, college degree or above (education), married (marital status), Han (ethnicity), monthly income ≥ ¥6,000, presence of chronic disease, drinker (alcohol consumption), smoker (smoking status), physically active, socially engaged, and having social support.

*p* < 0.05 was considered statistically significant.

## 5 Discussion

This study employed latent profile analysis (LPA) to model cognitive function profiles among community-dwelling and institutionalized older adults based on the five MMSE domains. The three-class model was selected as the optimal solution, balancing statistical fit with clinical interpretability. We minimized overfitting via a main-effects model, univariate screening, and EPV checks; findings remained after Holm adjustment. Although AIC, BIC, and aBIC values continued to decline as the number of classes increased, and both the LMRT and BLRT indicated significant improvement with additional classes (*p* < 0.01), the three-class model offered a more parsimonious and clinically meaningful classification. Given the cross-sectional design, all findings should be interpreted as associations rather than causal relationships; Odds ratios reflect odds latent-class/profile membership only and do not indicate causal effects. This finding aligns with previous studies, which emphasize model simplicity and interpretability when statistical indices conflict with theoretical rationale (Nylund et al., 2007). The entropy value increased from 0.823 in the two-class model to 0.891 in the three-class model, indicating improved classification accuracy and further supporting the suitability of the three-class solution (Tein et al., 2013). The final model classified participants into a high cognitive function group (41.2%), a moderate cognitive function group (48.2%), and a low cognitive function group (10.7%). These class proportions reflect the sample distribution and should not be interpreted as population prevalence. This classification pattern aligns with the multidimensional heterogeneity of the MMSE, suggesting that cognitive aging does not follow a single linear trajectory, but rather exhibits distinct subgroup variations (Clark, 2013).

The univariate analysis revealed significant differences in socio-demographic characteristics, health-related behaviors, and psychological status across cognitive function profiles, consistent with findings from previous studies (Dong et al., 2024).

Results from the multivariate logistic regression analysis further revealed a “health risk triad”—frailty, depression, and sleep disturbances—that were jointly associated with higher odds of belonging to lower cognitive-function profiles.

Although per-point OR may appear small, clinical change typically occurs over multi-point ranges or around validated cut-pointswithin such ranges the direction and cumulative impact remain meaningful at the population level. Thus, these associations are not only statistically significant but also clinically relevant. This finding aligns with previous studies (Liao et al., 2022; Ma et al., 2019; Shimada et al., 2014), and potential mechanisms are discussed as follows:

1. Frailty (OR = 1.075–1.244) was associated with lower cognitive profiles. Hypothesized pathways include interconnected physiological, psychological, and social pathways (Holland et al., 2024). Reductions in muscle mass, mitochondrial dysfunction, and chronic low-grade inflammation associated with frailty can increase blood–brain barrier permeability, triggering neuroinflammation and accelerating *β*-amyloid deposition. Declining physical function restricts social engagement and cognitive stimulation, while frailty-related reductions in self-efficacy and coexisting depressive tendencies may further amplify cognitive risk. These findings are consistent with Chen et al. (2023b), who emphasized the importance of frailty management as a key target in cognitive interventions for older adults.
2. Depression (OR = 1.059–1.191): Each 1-point increase in depression score was associated with 5.9–19.1% higher odds of lower cognitive profiles longitudinal evidence suggests possible bidirectional links. It is hypothesized that depression may contribute to cognitive decline via multiple neurobiological mechanisms, including reduced brain-derived neurotrophic factor (BDNF) levels, hippocampal atrophy, impaired neuroplasticity, and disrupted executive and memory functions. The association between depression and cognitive decline is supported by a recent longitudinal cohort study showing that higher depressive symptoms are associated with steeper subsequent memory decline (Yin et al., 2024). This study further reported that older adults with greater baseline depressive symptoms experienced a significantly faster rate of memory decline over time, with a reciprocal association also observed whereby accelerated memory decline was linked to a worsening of depressive symptoms, underscoring the bidirectional plausibility of this association.
3. Sleep disturbances, as reflected by higher PSQI scores (OR = 1.088), were associated with higher odds of belonging to the Moderate/Low cognitive profiles; Hypothesized mechanisms involve impaired *β*-amyloid clearance, neuroinflammation, and disrupted neural homeostasis (Liao et al., 2022).
4. Anxiety (inverse association within the non-clinical range) A one-standard-deviation increase in the standardized anxiety scores was associated with significantly lower of being classified into the Moderate or Low cognitive function group (OR = 0.979 and 0.977, respectively; both *p* < 0.001, reference group = high). within the non-clinical range (SAS < 50), higher levels of anxiety may be linked to better cognitive functioning. One possible explanation is that mild anxiety enhances vigilance and attentional engagement, which may transiently support cognitive performance (Yang et al., 2024). However, the current model assumes a linear relationship between anxiety scores and the logit of cognitive status and thus cannot evaluate potential non-linear associations such as an inverted U-shaped pattern. Future studies are encouraged to incorporate non-linear modeling strategies and longitudinal designs to explore the temporal and causal dynamics of this relationship.
5. Education and income. Education level and household income demonstrated dose–response associations with cognitive profiles. The lower the educational attainment, the higher the odds of belonging to the Low profile, with uneducated individuals showing an OR ranging from 8.042 to 24.702. These findings support the cognitive reserve hypothesis (Rodriguez et al., 2021). Moreover, participants in the middle-income category (¥2,000–4,999) had lower odds versus ≥¥6,000. These patterns are consistent with the hypothesis that economic status may influence cognitive health through enhanced health awareness, better access to medical care, and more proactive health behaviors.
6. Social support and behavioral patterns: group-specific associations with cognitive profiles. In our study, social support was identified as a protective factor for cognitive function in older adults (OR = 0.530–0.696), i.e., an inverse association (adequate social support, Yes vs. No), with the protective association particularly evident among individuals receiving both adequate material and emotional support. Participants with high levels of social support were significantly less likely to be classified into the moderate cognitive function group (OR = 0.696) or the low cognitive function group (OR = 0.530; both *p* < 0.001; reference group = High), indicating a clear inverse association in this cross-sectional analysis. This finding is consistent with previous research (Kelly et al., 2017; Mogic et al., 2023). Mechanistically, material and emotional support may relate to greater social participation and exposure to cognitively stimulating activities, potentially supporting cognitive resilience in later life. Given the cross-sectional design, these results indicate associations rather than causal effects.
7. Gender and Age: Male participants had a significantly lower odds of belonging to the Moderate/Low cognitive profiles compared to females (OR = 0.556–0.876), in this cross-sectional analysis,which may be partly related to levels of social engagement. Additionally, individuals aged 60–79 were more likely to be in the higher cognitive profile than those aged ≥80, consistent with the well-established pattern of age-related cognitive decline. This finding on the role of gender and age is consistent with previous epidemiological research (Delpak and Talebi, 2020).
8. Subgroup differences in marital status revealed that being unmarried, divorced, or widowed was associated with higher odds of belonging to the Low cognitive profile. This finding is consistent with previous evidence indicating that the absence of marital support is associated with greater cognitive vulnerability (Yanxue et al., 2023; Yuchao et al., 2024). Spousal companionship and health-related interactions may provide emotional and practical support that potentially supports cognitive functioning. In contrast, individuals without spouses—particularly those who are widowed or divorced—may experience disrupted social networks, loneliness, depressive symptoms, and reduced engagement in health-promoting behaviors, all of which may be linked to poorer cognition. The impact of marital loss may be particularly pronounced in the low cognitive profile, possibly due to diminished physiological reserve and limited personal resources, underscoring the need to prioritize support. By comparison, individuals in the Moderate cognitive profile—who typically have higher education levels and stronger social engagement—may buffer the effects of marital loss through alternative sources of social support. Future longitudinal studies are needed to clarify temporal ordering and the causal pathways between marital status and cognitive decline, and to inform the development of tailored, stratified intervention strategies (Liu et al., 2019).
9. Lack of social participation was associated with higher odds of being categorized in the low cognitive latent category (OR = 1.880), whereas being socially active was associated with lower odds. This finding provides indirect, correlational support for the proposition that community-based social interventions respond to cognitive deterioration in older adults (Jun-hong et al., 2023); however, given the cross-sectional design, the present study only demonstrates a statistical association, which cannot be interpreted causally and does not constitute direct evidence of intervention effectiveness (Jun-hong et al., 2023).
10. The associations of smoking and alcohol consumption on cognitive function varied across cognitive groups profiles, indicating notable intergroup heterogeneity. Among individuals classified into the Moderate cognitive profile, non-smokers (OR = 1.214) and non-drinkers (OR = 1.320) showed higher odds of cognitive membership (vs the High profile). This pattern, consistent with earlier studies (Ge et al., 2020; Wu et al., 2019), may reflect reverse causality—individuals with early cognitive decline modify or cease such behaviors, rather than smoking or drinking being inherently protective (Sabia et al., 2012). Other studies have suggested that the neurotoxic effects of smoking may manifest later and be obscured by comorbidities during more advanced stages of frailty (Durazzo et al., 2017). The relationship between moderate alcohol consumption and cognitive performance remains controversial: while some studies have reported enhanced cognitive function among moderate drinkers (Richard et al., 2017; Sun et al., 2011), other research has found inconsistent or contradictory results regarding both alcohol use and smoking (Jin et al., 2021). Future longitudinal studies are needed to disentangle temporal ordering and test causal pathways and clarify the timing and directionality of these associations.

## 6 Limitations

The present study also has several limitations. First, the cross-sectional design was not able to infer causality or characterize individual cognitive change over time; a longitudinal design incorporating biomarkers could be used in the future. Second, there may be ceiling/floor effects and education/culture-related differences in the MMSE, which may reduce sensitivity to mild differences, although five dimensional z-scores and education-stratified cutoffs were used to mitigate the effects. Again, latent profile analysis (LPA) is sensitive to sample size and indicator selection, and there may be inconsistencies between fitted indicators; a three-category scheme was chosen for this study to balance statistical fit with clinical interpretability. Despite the adequacy of EPV, the possibility of overfitting or modeling bias cannot be completely excluded. In addition, participants came from 6 communities and 9 long-term care facilities in the same region, and individuals were nested in field sites without specific field clustering corrections, potentially biasing the standard errors small; applicability to other regions or populations requires caution. Exclusion criteria for feasibility may introduce a healthier participant bias, with results more applicable to stable community/institutionalized populations; some variables were derived from self-reports, with possible recall and social desirability bias; relevant laboratory indicators were also lacking in this study. Future research suggests multicenter longitudinal follow-up, incorporating educational corrections or supplemental scales, and using mixed-effects/clustering robust methods with more objective measures to further validate and extend the findings of this study.

## 7 Conclusion

This study identified three latent cognitive function profiles in older adults based on MMSE scores, demonstrating substantial intergroup heterogeneity. Frailty, depressive symptoms, and poor sleep quality emerged as primary risk factors for cognitive impairment, whereas social support, higher educational attainment, and moderate income levels were associated with protective effects.

## Data Availability

The raw data supporting the conclusions of this article will be made available by the authors, without undue reservation.
